# Challenges of Secondary Glaucoma Management Following Congenital Cataract Surgery, Penetrating Keratoplasty and Vitreoretinal Surgery

**DOI:** 10.3390/diagnostics14080837

**Published:** 2024-04-18

**Authors:** Valeria Coviltir, Maria Cristina Marinescu, Miruna Gabriela Burcel, Maria-Emilia Cerghedean-Florea, Adrian Hașegan, Ciprian Tănăsescu, Mihaela Laura Vică, Horațiu Dura

**Affiliations:** 1 Faculty of Medicine, “Carol Davila” University of Medicine and Pharmacy, 050474 Bucharest, Romania; 2 Clinical Hospital of Ophthalmologic Emergencies, 010464 Bucharest, Romania; 3 City Hospital Videle, 145300 Videle, Romania; 4 Faculty of Medicine, “Lucian Blaga” University of Sibiu, 550024 Sibiu, Romania; 5 Department of Cellular and Molecular Biology, “Iuliu Haţieganu” University of Medicine and Pharmacy, 400012 Cluj-Napoca, Romania; 6 Institute of Legal Medicine, 400006 Cluj-Napoca, Romania

**Keywords:** congenital cataract, secondary glaucoma, Ahmed valve

## Abstract

Glaucoma is one of the world’s leading causes of irreversible vision loss. It is often asymptomatic until it reaches an advanced stage, which can have a significant impact on patients’ daily lives. This paper describes the case of a 50-year-old female patient who presented with acute onset of ocular pain, photophobia, and loss of visual acuity in her right eye (RE). The patient’s medical history includes congenital cataracts, surgical aphakia, nystagmus, strabismus, amblyopia, and secondary glaucoma. Ophthalmological examination showed BCVA RE-hand movement, left eye (LE)—0.08 with an intraocular pressure (IOP) of 30 mmHg in RE and 16 mmHg in LE. Biomicroscopic examination of RE showed corneal graft, epithelial and endothelial edema, endothelial precipitates, corneal neovascularization, aphakia, and Ahmed valve superotemporally. Despite maximal topical and systemic treatment, Ahmed valve, and trabeculectomy, secondary glaucoma in the right eye remained refractory. Reimplantation of an Ahmed valve was performed. This resulted in a favorable outcome with increased visual acuity and controlled intraocular pressure. The combination of aphakia, penetrating keratoplasty, and secondary glaucoma is a challenge for any surgeon. It is important that both the perioperative risks and the possible complications are carefully assessed in each patient, especially if associated pathology is present.

**Figure 1:**
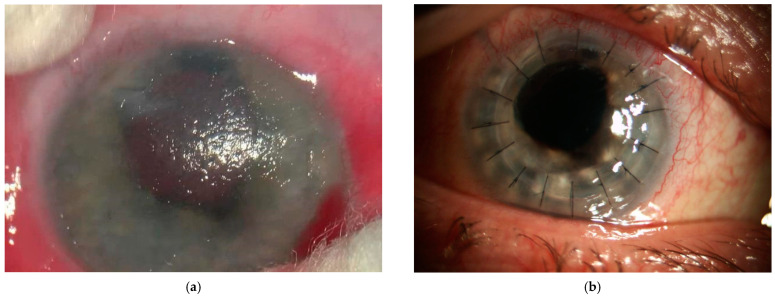
(**a**) Slit-lamp photograph of the bullous keratopathy in the right eye; (**b**) postoperative aspect of the right eye 1 day after penetrating keratoplasty. Congenital cataract is a disorder of lens transparency present at or shortly after birth. It is a major cause of blindness or treatable visual impairment in children. There are many causes of congenital cataracts. These include intrauterine infections, exposure of the pregnant woman to radiation, medication during pregnancy, as well as genetic and metabolic causes. Cataract surgery may be recommended for preverbal children who have dense cataracts, strabismus, or nystagmus. Some studies have suggested that performing cataract surgery within the first four weeks of life may increase the risk of developing aphakic glaucoma. As a result, it is generally advised to delay the surgery until the patient is at least 28 days old [1]. According to research, between 30% and 50% of congenital cataracts are caused by genetic mutations affecting the proteins in the lens structure. Congenital cataracts are often caused by single-gene disorders. Autosomal dominant inheritance is widely considered to be the most common mode of congenital cataracts. Currently, over 34 loci and 18 genes on different chromosomes have been identified to be associated with autosomal dominant congenital cataracts (ADCC) [2]. A 50-year-old female patient was referred from an outpatient clinic for secondary glaucoma, which was refractory to treatment. The patient has a history of congenital cataracts in both eyes, which were removed when she was one year old, and secondary aphakia. The patient has no systemic conditions, does not take any general medication, and has no family history related to ocular pathologies. Although the most common forms of adult cataracts are age-related, the occurrence of congenital cataracts is often associated with other eye disorders [3]. She was diagnosed with amblyopia in the left eye (LE) and nystagmus in both eyes. The patient underwent surgeries for strabismus in both eyes at the age of 3 (RE) and 5 (LE). She was diagnosed with glaucoma following congenital cataract surgery at age 24 and prescribed topical hypotensive medication. After 9 years, at the age of 35, the glaucoma was no longer properly controlled with maximal topical medication. A bilateral Ahmed valve implantation was performed due to the risk of scarring and bleb failure with a trabeculectomy. Glaucoma is the leading cause of irreversible visual loss and the second most common cause of blindness, leading to a huge burden worldwide [4,5]. Congenital glaucoma commonly occurs among communities that encourage consanguinity and may be classified as primary (without any ocular or systemic developmental anomalies) or secondary congenital glaucoma (accompanied by other pathologies) [6,7,8]. It is characterized by retinal progression loss of ganglion cells, which leads to changes in the optic nerve head and visual field defects, affecting visual quality of life [9]. Aphakic glaucoma is the second most common cause of glaucoma in the pediatric population, being classified as a secondary form of open-angle glaucoma [10]. The development of aphakic glaucoma may be multifactorial. Various risk factors have been identified, including age at the time of lensectomy, type of cataract, IOL implantation, primary posterior capsulotomy with anterior vitrectomy, preexisting ocular abnormalities, additional intraocular surgery, and family history of congenital cataract. The pathophysiological mechanisms of aphakic glaucoma can be classified into two groups: angle closure and open angle. It is believed that angle-closure glaucoma may be caused by an intense postoperative inflammatory response, which can result in synechia in the chamber angle or pupillary block. The mechanism underlying open-angle glaucoma is poorly understood and thought to be multifactorial. Chemical and mechanical hypotheses have been proposed [11]. At the age of 48, the patient was referred to a corneal transplant center with a diagnosis of right eye bullous keratopathy (as shown in Figure 1a). In January 2018, a penetrating keratoplasty (PRK) was performed (Figure 1b). Following penetrating keratoplasty (PKP), the risk of developing glaucoma is significant: different studies report an incidence of 9–31% early after surgery and 18–35% within the late postoperative period [12]. The etiology for this disorder is multifactorial; risk factors contributing to ocular hypertension (OHT) and glaucoma after PKP include preexisting glaucoma, combined surgical procedures, corneal perforation, previous PKP, steroid response, suturing technique, and the diameter of the graft. In addition, the change in anterior chamber angle structure is suspected to be related to the incidence of glaucoma after PKP [13]. A clear understanding of the various mechanisms that operate during different time frames following PK is essential to chalk out the appropriate management algorithms [14]. Wherever indicated, prompt therapy should be initiated to lower intraocular pressure and salvage vision [15]. The surgery was performed without intraoperative complications; the tube remained in a good position without needing replacement. The patient was followed closely and, on the second day, postoperatively, was diagnosed with choroidal detachment (Figure 2a). Suprachoroidal hemorrhage is a rare but potentially devastating complication that can occur during or after penetrating keratoplasty [16]. The risk of occurrence is low, between 0.5 and 1%, and one of the predisposing factors is previous eye surgery [17,18]. It is important for surgeons to be aware of the potential risk of suprachoroidal hemorrhage during eye surgeries and to take steps to minimize this risk, such as carefully monitoring intraocular pressure and avoiding excessive manipulation of the eye. Early detection and prompt treatment are crucial in preventing long-term complications. One month after the PKP, the patient presented a sudden decrease in visual acuity in her right eye, and upon examination, she was diagnosed with rhegmatogenous retinal detachment (Figure 2b).

**Figure 2:**
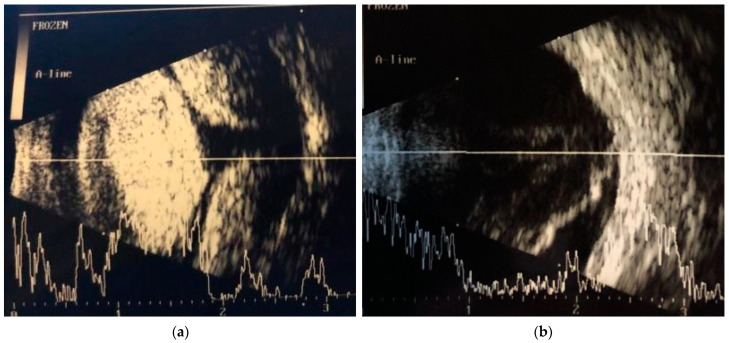
(**a**) RE echography revealing choroidal detachment 1 day after PKP; (**b**) RE echography revealing retinal detachment 1 month after PKP. B-scan ocular ultrasound of the RE one day after PRK shows a choroidal detachment, which was treated medically and then surgically drained. However, 1 month after the PKP, she was diagnosed with rhegmatogenous retinal detachment with a horseshoe retinal break inferonasally. Subsequently, she underwent a pars plana vitrectomy 23 Ga with silicone oil, and the retina was reattached. The silicon oil extraction was carried out 2 months after the vitrectomy. Postoperatively, the retina remained attached, but the intraocular pressure (IOP) increased to approximately 30 mmHg under maximal treatment, including systemic carbonic anhydrase inhibitor (CAI). The surgical management of retinal disorders can lead to short-term elevations in intraocular pressure (IOP) and, ultimately, long-term glaucomatous damage if not treated in a timely manner [19]. Elevated IOP is a potentially serious complication following vitreoretinal surgery for a number of reasons. Mechanisms could include secondary pupillary block, preexisting glaucoma [20], chronic inflammation [21], silicone oil migration into the anterior chamber [22], trabecular meshwork obstruction caused by silicone emulsion [23], rubeosis iridis [24], or iridocorneal angle closure caused by anterior synechiae [25,26]. Identifying the mechanism that causes the raised IOP is crucial as the management of glaucoma can differ accordingly [27]. In our case, it was decided that the patient would need another antiglaucomatous surgery, and RE trabeculectomy was performed. Postoperatively, IOP was stabilized in the range of 16–22 mmHg under topical treatment, with fluctuations. Unfortunately, the patient’s condition worsened three months after the trabeculectomy. The patient reported a decrease in visual acuity and pain in the right eye, which began three days before presenting to our clinic. The slit-lamp examination of the anterior segment of the right eye is described below (Figure 3). The left eye presented with a clear cornea, oval pupil, deep anterior chamber, and aphakia. IOP was 30 mmHg in RE and 12 mmHg in LE in both eyes with maximal treatment: topical CAI twice daily (BID), fixed combination beta-blocker and prostaglandin analog once daily (QD), and systemic CAI QD.

**Figure 3:**
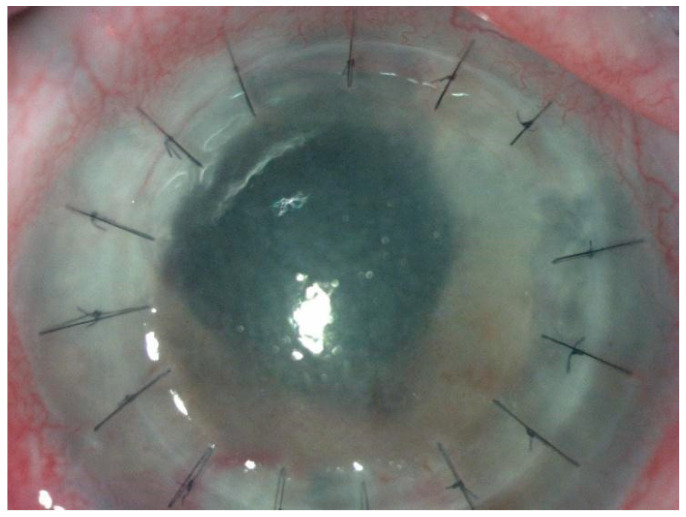
Slit-lamp examination of the right eye at first presentation in our clinic shows a corneal graft with separated sutures, epithelial and endothelial edema, endothelial precipitates, corneal neovascularization superiorly (host cornea), irregular pupil, photomotor reflex absent, deep anterior chamber, aphakia, Ahmed valve superotemporally, with siliconic tube visible at 11 o‘clock. Ophthalmological examination revealed visual acuity of hand movement, perception of light present in all 4 quadrants in RE, best corrected visual acuity (BCVA) of 0.08 in LE, IOP of 40 mmHg RE, and 16 mmHg LE. A gonioscopy could not be performed. Fundus examination showed a red reflex in the RE, but details cannot be differentiated, and the LE showed an oval, pale optic nerve head with peripapillary atrophy and without further details. Specular microscopy was performed: the endothelial cell density could not be measured in RE and was 2237 cell/mm^2^ in LE. Corneal pachymetry showed an average central corneal thickness of 743 μm in RE and 582 μm in LE. The visual field examination revealed severe constriction in both eyes. Autorefractometry revealed high hyperopia RE, moderate hyperopia, and compound hyperopic astigmatism LE. It was decided that the surgical management of the case should involve a secondary Ahmed valve implantation (Figure 4).

**Figure 4:**
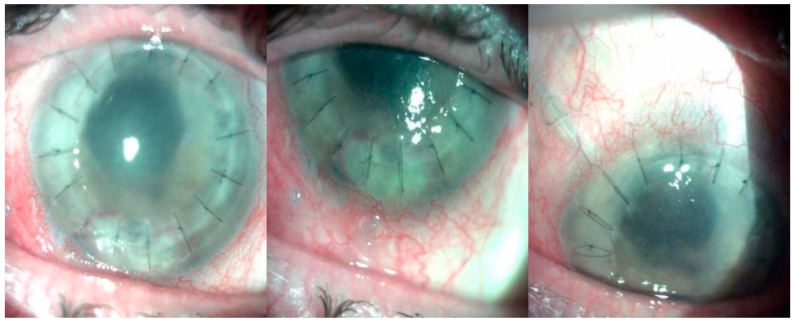
Slit-lamp photographs after the second valve Ahmed implantation revealed a decrease in corneal edema. The surgery was performed without complications: the second valve was implanted in the inferotemporal quadrant, and the silicon tube was placed in the posterior chamber. Postoperative treatment was topical nonsteroidal anti-inflammatory and fixed combination broad-spectrum antibiotic and steroidal anti-inflammatory. The B-Scan ultrasonography showed an attached retina without choroidal detachment. At two weeks follow-up, BCVA RE increased to counting fingers at 1 m, light perception positive in all 4 quadrants. IOP decreased to 14 mmHg. The risk of developing glaucoma after congenital cataract surgery is higher if the patient was initially diagnosed with a nuclear or total cataract, underwent surgery before the age of one, had postoperative complications or cycloplegic use, or had a corneal diameter of less than 10 mm [28]. Mechanisms proposed in glaucoma after congenital cataract surgeries are of chemical or mechanical nature. Firstly, in the absence of the lens, a vitreous chemical component may have access to the trabecular meshwork, resulting in damage. Secondly, glaucoma may emerge secondary to the lack of mechanical support given to the trabecular meshwork by the intraocular lens (IOL). Furthermore, lens extraction early in life disrupts the normal development of iridocorneal angle elements [29]. Statistically, the incidence of glaucoma is higher in aphakic eyes than in phakic eyes in the pediatric population. Research has presented possible explanations for the apparent protective effect of intraocular lens implantation; however, selection bias may play a pivotal role, as the ophthalmologist is more likely to choose aphakia in a case with glaucoma risk factors and signs [30]. Glaucoma is an important complication of keratoplasty, and aqueous shunts can be an effective option for managing intraocular pressure (IOP) in these cases [31]. On the other hand, implantation of an aqueous shunt increases the risk of corneal decompensation and graft failure in the case of a tube implanted in the anterior chamber [32]. Several mechanisms contribute to this: endothelial trauma due to surgical manipulation and cornea-tube contact postoperatively; the formation of fibrosis and anterior synechiae around the tube; and the blood-aqueous barrier breakdown, facilitated by the shunt, which may accelerate the immune graft rejection [33]. These phenomena may be avoided through a pars plana insertion of the aqueous shunts [34]. Performing an aqueous shunt implantation at the same time as the PKP is indicated in cases of known or anticipated glaucoma, which, most likely, would be properly controlled with hypotensive medication [35]. Our patient has developed glaucoma 23 years after congenital cataract surgery. Initially, she was prescribed topical medication. When the IOP was not properly controlled, an Ahmed valve implantation was decided. According to the literature, surgery is required in 27–83% of cases. Angle surgery is one of the methods investigated in the treatment of glaucoma following cataract surgery. The available data on the outcomes of this treatment modality are limited and have mostly been presented by small retrospective cohorts. Success rates have been variably reported, from 16% to 93% [36]. Results of trabeculectomy in eyes with glaucoma following cataract surgery are generally poor and report a high failure rate, with 50% requiring two or more surgeries to control IOP. Ultimately, these children may need tube implants to control IOP [37]. Pakravan et al. reported a 90% success rate at one year following glaucoma drainage device implantation as a primary procedure in eyes with glaucoma following cataract surgery. After five years of follow-up, the success rate was 72% [38]. Regarding literature studies about second valve implantation, Becerril-Cazadero R et al. reported that the addition of a second AGV has been found to be effective in reducing IOP by approximately 47% over a mean survival time of 15 months (13.7 to 16.5), with a success rate of 60% [39]. In conclusion, performing glaucoma surgery on patients with aphakia and penetrating keratoplasty can be challenging for any surgeon. In this case, the prognosis is reserved due to age, multiple eye conditions, and multiple eye surgeries. An interesting aspect of this case is the potential future evolution of the eye, including the long-term control of intraocular pressure (IOP) following multiple surgeries and the possibility of another penetrating keratoplasty. The worst nightmare could be needing additional glaucoma surgery.

## Data Availability

All relevant data have been presented in this manuscript, and further inquiry can be directed to the corresponding author.
